# Supplementary figures and images for: Stc2a inhibits IGF-stimulated somatic growth in favor of organismal survival under hypoxic stress
Source: Front Endocrinol (Lausanne). 2026 Jan 7;16:1729649. doi: 10.3389/fendo.2025.1729649 (PMC12819173; doi:10.3389/fendo.2025.1729649)

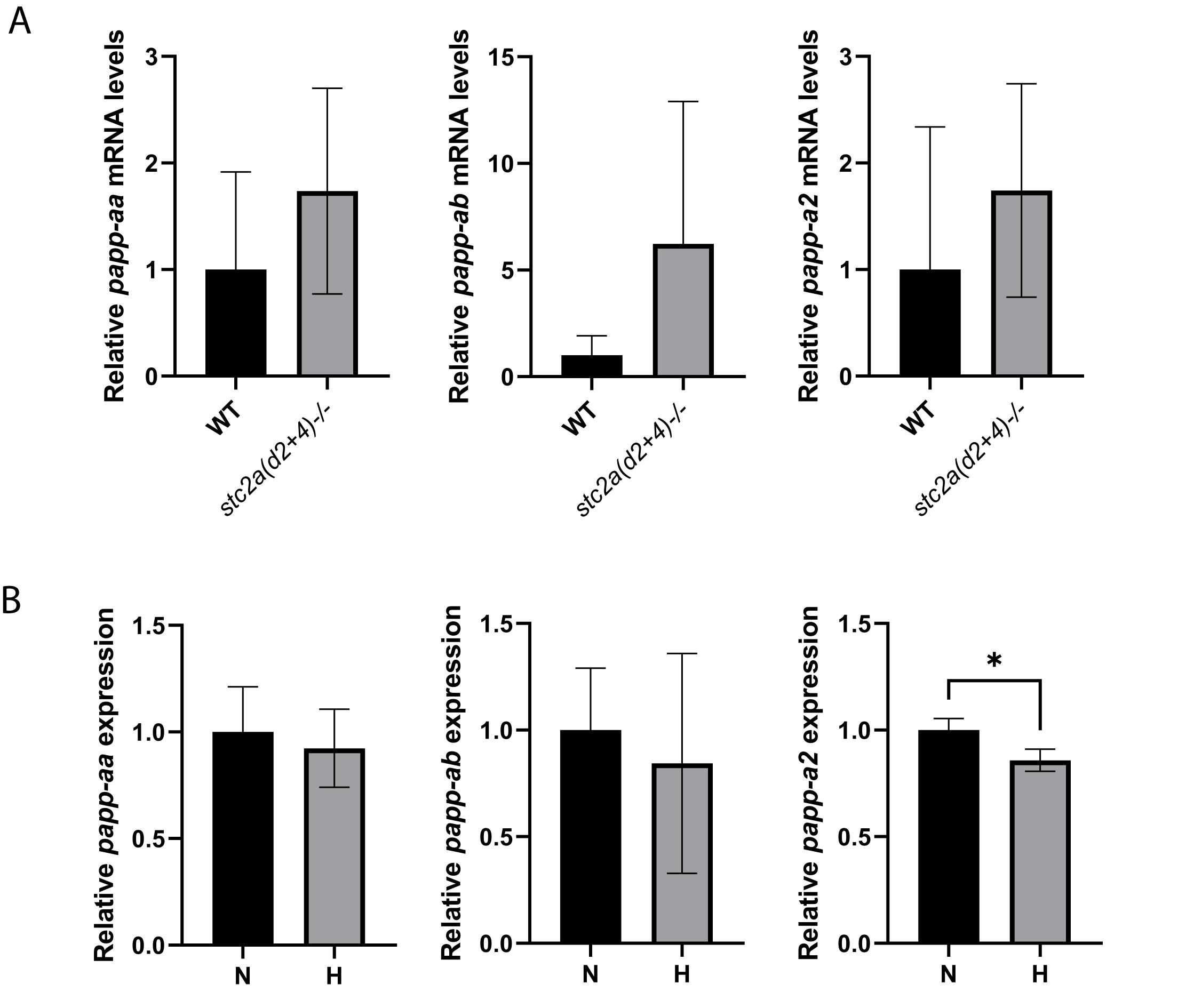

Supplement: Supplementary Figure 1 — (A) Principal component analysis (PCA) plot of normoxia (N) and hypoxia (H) groups. The plot shows the first two principal components (PC1 and PC2) of normalized gene expression data, illustrating the overall variance and separation between normoxia and hypoxia groups. (B) The mRNA levels of the indicated genes were measured by qPCR (blue) or RNA-seq (red) and shown as the ratio between the hypoxia groups and the normoxia groups. n = 2-4. (C, D) Gene-concept networks (CNETs) of differentially expressed genes. CNETs show the top five significantly enriched GO molecular function terms for (C) up-regulated and (D) down-regulated DEGs. Small nodes represent genes associated with each GO term, with node color indicating fold change, whereas large cyan nodes represent the GO terms. [file Image1.tif]

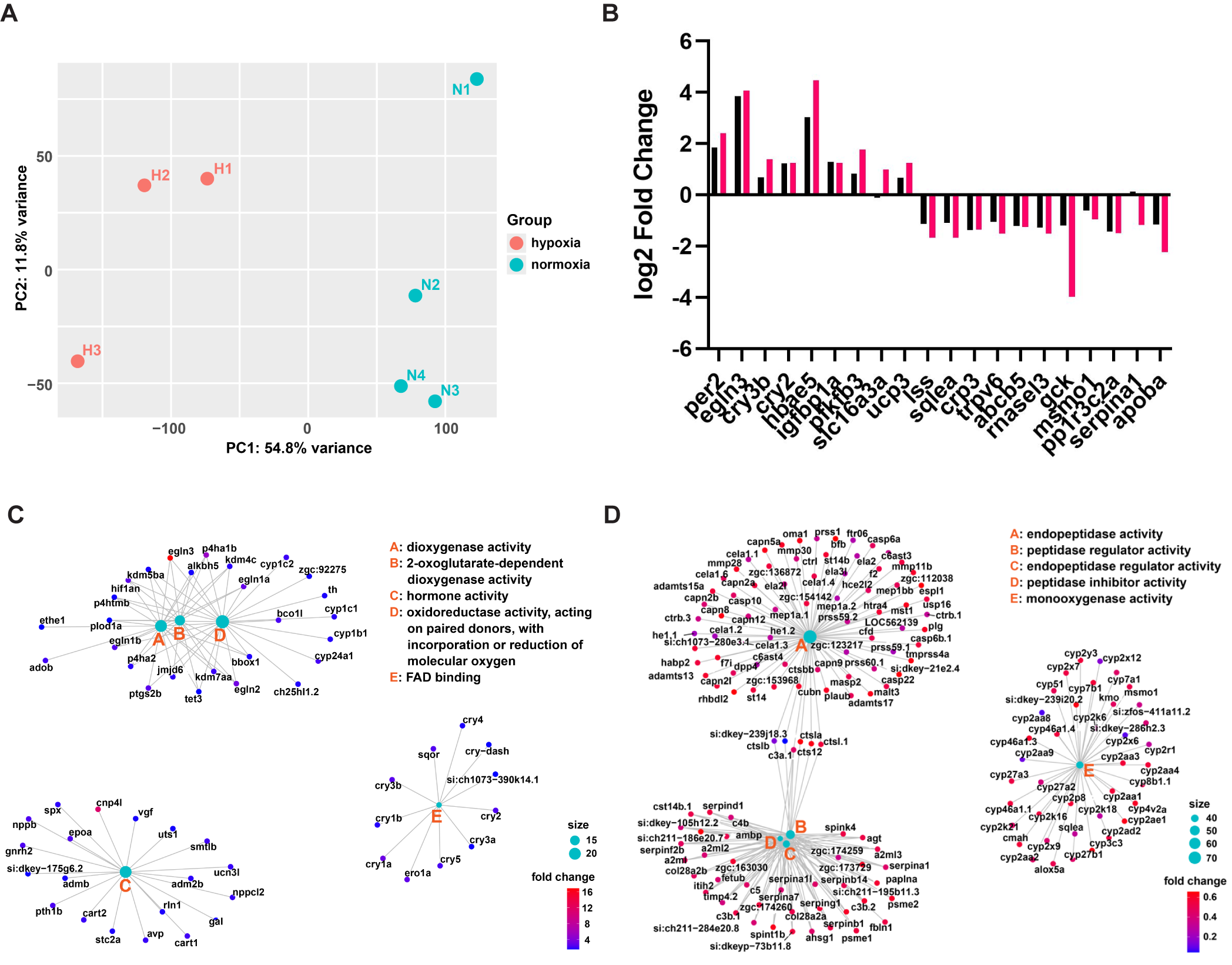

Supplement: Supplementary Figure 2 — (A) Schematic diagram of zebrafish stc2a gene and the engineered mutants. Boxes represent exons and lines represent introns. Filled boxes represent protein coding regions and open boxes represent untranslated regions. The PAM motif is represented in blue color. Dashed lines indicate conserved cysteine residues. Blue boxes indicate N-glycosylation sites. (B) qRT-PCR measurement of stc2a mRNA levels in 5 dpf wild-type (WT) and stc2a(Δ5)-/- fish. **, p < 0.01. (C) qRT-PCR measurement of stc2a and stc2b mRNA levels in 5 dpf wild-type (WT) and stc2a(Δ2 + 4)-/- fish. ***p < 0.001. (D) qRT-PCR measurement of stc2a mRNA levels in adult wild-type and stc2a(Δ2 + 4)-/- fish. [file Image2.tif]

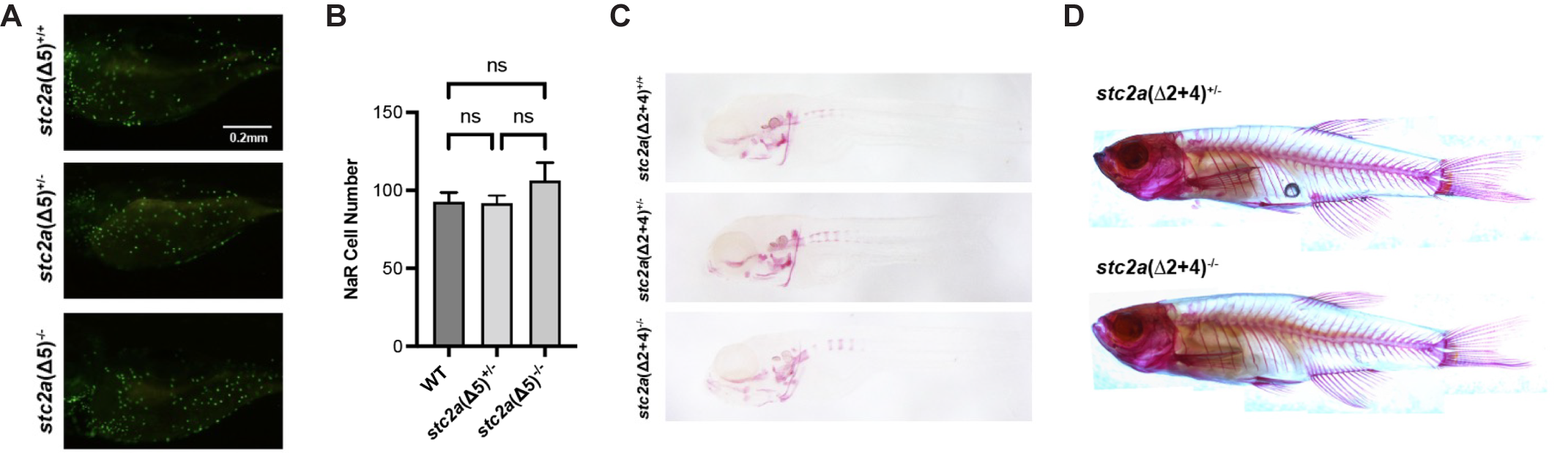

Supplement: Supplementary Figure 3 — Loss of Stc2a does not change ionocyte cell proliferation or bone mineralization. (A, B) NaR cell number of the indicated genotype fish in the Tg(igfbp5a:GFP) background were measured at 5 dpf. Representative images of GFP-expressing NaR cells are shown in (A) and quantified results in (B). Data are shown as mean ± SEM. n = 5-18, ns, not statistically significant, one-way ANOVA followed by Tukey’s multiple comparisons test. (C) Representative images of 7 dpf fish of the indicated genotypes stained by Alizarin red. (D) Representative images of 1 year-old adult fish stained by Alizarin red. [file Image3.tif]

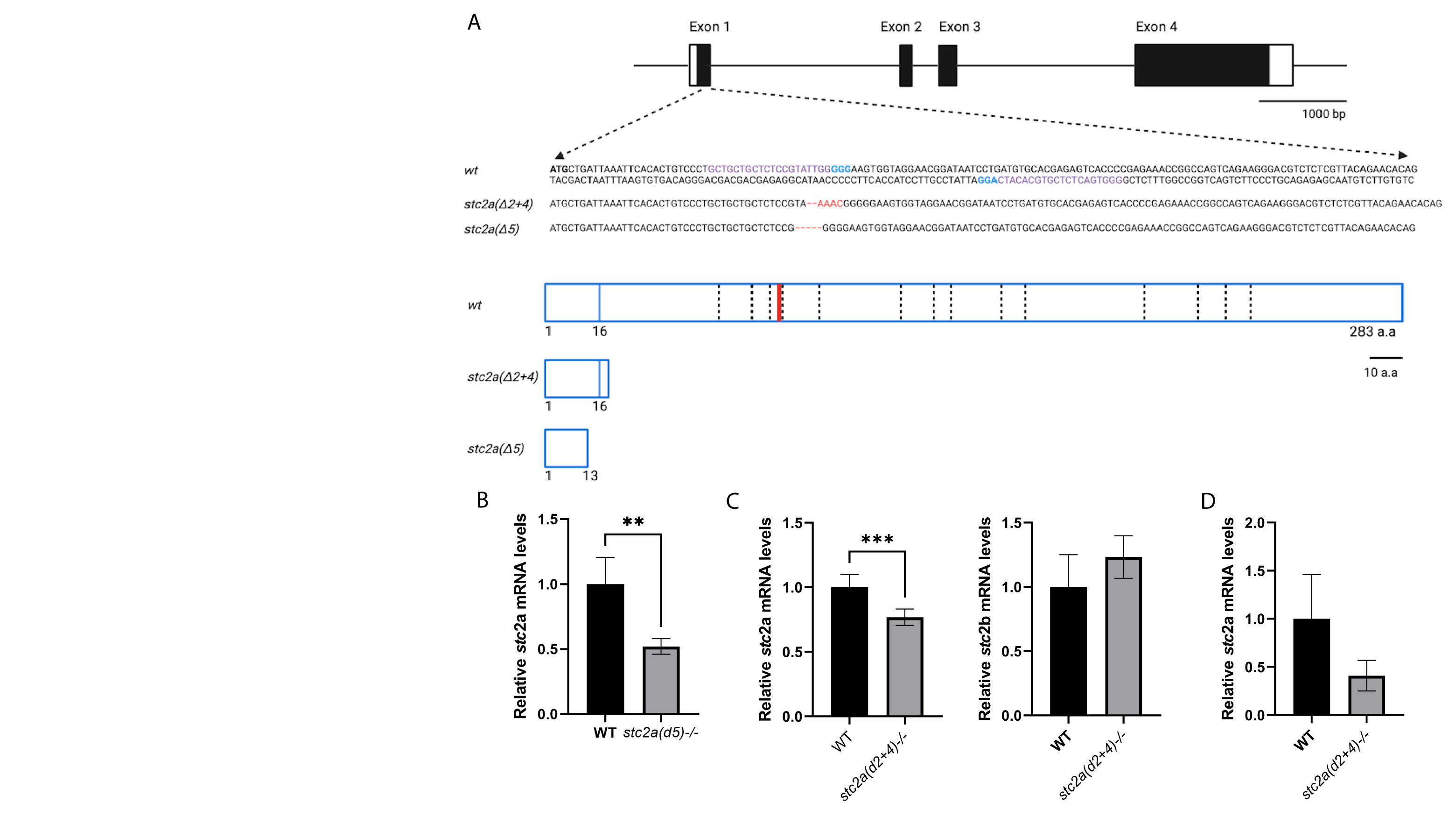

Supplement: Supplementary Figure 4 — (A) Loss of Stc2a does not change papp-aa, papp-ab, and papp-a2 mRNA levels. qRT-PCR results of 5 dpf fish larvae of the indicated phenotypes. Data are shown as mean ± SEM. n = 4, No statistical significance was detected. (B) Effect of hypoxia on papp-aa, papp-ab, and papp-a2 mRNA levels. Relative mRNA abundance is extracted from RNA-seq data set (TPM) and calculated and shown as relative change of the control group. *p <0.05. [file Image4.tif]
